# Left Ventricular Geometry and Blood Pressure as Predictors of Adverse Progression of Fabry Cardiomyopathy

**DOI:** 10.1371/journal.pone.0140627

**Published:** 2015-11-23

**Authors:** Johannes Krämer, Bart Bijnens, Stefan Störk, Christian O. Ritter, Dan Liu, Georg Ertl, Christoph Wanner, Frank Weidemann

**Affiliations:** 1 Department of Medicine I, Cardiology Unit, University of Würzburg, Würzburg, Germany; 2 Department of Pediatrics and Adolescent Medicine, University of Ulm, Ulm, Germany; 3 Department of Medicine I, Nephrology Unit, University of Würzburg, Würzburg, Germany; 4 ICREA–Universitat Pompeu Fabra, Barcelona, Spain and KU Leuven, Leuven, Belgium; 5 Comprehensive Heart Failure Center, University of Würzburg, Würzburg, Germany; 6 Department of Radiology, University of Würzburg, Würzburg, Germany; 7 Department of Internal Medicine II, Katharinen-Hospital Unna, Unna, Germany; KRH Robert Koch Klinikum Gehrden, GERMANY

## Abstract

**Background:**

In spite of several research studies help to describe the heart in Fabry disease (FD), the cardiomyopathy is not entirely understood. In addition, the impact of blood pressure and alterations in geometry have not been systematically evaluated.

**Methods:**

In 74 FD patients (mean age 36±12 years; 45 females) the extent of myocardial fibrosis and its progression were quantified using cardiac magnetic-resonance-imaging with late enhancement technique (LE). Results were compared to standard echocardiography complemented by 2D-speckle-tracking, 3D-sphericity-index (SI) and standardized blood pressure measurement. At baseline, no patient received enzyme replacement therapy (ERT). After 51±24 months, a follow-up examination was performed.

**Results:**

Systolic blood pressure (SBP) was higher in patients with vs. without LE: 123±17 mmHg vs. 115±13 mmHg; P = 0.04. A positive correlation was found between SI and the amount of LE-positive myocardium (r = 0.51; P<0.001) indicating an association of higher SI in more advanced stages of the cardiomyopathy. SI at baseline was positively associated with the increase of LE-positive myocardium during follow-up. The highest SBP (125±19 mmHg) and also the highest SI (0.32±0.05) was found in the subgroup with a rapidly increasing LE (ie, ≥0.2% per year; n = 16; P = 0.04). Multivariate logistic regression analysis including SI, SBP, EF, left ventricular volumes, wall thickness and NT-proBNP adjusted for age and sex showed SI as the most powerful parameter to detect rapid progression of LE (AUC = 0.785; P<0.05).

**Conclusions:**

LV geometry as assessed by the sphericity index is altered in relation to the stage of the Fabry cardiomyopathy. Although patients with FD are not hypertensive, the SBP has a clear impact on the progression of the cardiomyopathy.

## Introduction

Fabry disease (FD) is an X-linked lysosomal storage disorder caused by a deficiency of α-galactosidase A. The clinical presentation is a multi-systemic disease affecting kidneys, nervous system and the heart. [1, 2] The cardiac pathophysiologic correlate is the accumulation of globotriaosylceramides in cells, especially in myocytes, causing left ventricular (LV) hypertrophy thus inducing myocardial replacement fibrosis. [3, 4, 5, 6] The main cardiac manifestations are arrhythmias [7] and heart failure, which are also responsible for reduced life expectancy in FD. [8, 9]

As main drivers for the development of the Fabry cardiomyopathy, the storage of globotriaosylceramides in myocytes [10] with subsequent LV hypertrophy [3] and the typical feature of myocardial fibrosis [11] have been well investigated. The reason for development of fibrosis in basal posterolateral wall segments remains unclear. It can be speculated that this might be caused by different pressure and wall stress conditions in the different wall segments, which would imply, that a combination of extrinsic and intrinsic factors might lead to cell destruction and replacement fibrosis. However, changes in LV geometry and loading conditions as extrinsic factors, and their impact on the myocardium and disease progression were not studied so far.

An easy, non-invasive tool for the assessment of LV geometry is the 3D sphericity index. [12] This index is calculated by standard echocardiographic parameters and provides information about the shape of the LV. It was evaluated in mitral regurgitation [13], dilated cardiomyopathy [14], hypertensive heart disease [15] and myocardial infarction [16]. It was not yet used as a prognostic marker. Structural damage of the LV myocardium may adversely alter LV geometry; in addition, altered LV geometry by itself might further induce LV structural damage by changing the pressure conditions and thereby adversely impact on the progression of the Fabry cardiomyopathy.

In general, a (very) high blood pressure is not considered typical for Fabry patients. [17, 18, 19] It is well acknowledged, however, that a slight increase in blood pressure already significantly alters LV loading conditions (especially afterload), thus potentially accelerating the development and progression of the cardiomyopathy.

The aim of this study was to explore links between the severity and progression of the Fabry cardiomyopathy and geometrical changes of the LV myocardium, as well as blood pressure. Additionally, predictors for progression of the cardiomyopathy were investigated. We hypothesized that standard echocardiography can detect geometry changes of the LV and that an altered geometry, in association with the blood pressure, is connected with replacement fibrosis and progression of the cardiomyopathy. To understand the interaction of these factors would greatly enhance our concepts regarding the clinical assessment and follow-up of patient with Fabry cardiomyopathy.

## Materials and Methods

### Study population

In total, 162 consecutive FD patients were screened at their first visit at the Fabry center Wuerzburg. No patient used enzyme replacement therapy (ERT). Criteria for inclusion were (1) genetically proven Fabry disease, (2) feasibility of cardiac magnetic resonance imaging (cMRI) with contrast agent, (3) feasibility of echocardiography, (4) absence of coronary artery disease, and (5) informed consent for the examinations and participation in the study. Finally, 74 patients (45 female) were included in this study. Reasons for exclusion (n = 88) were: contraindications for cMRI, such as claustrophobia or pacemakers; stage 4 or 5 renal dysfunction; uncontrolled hypertension (SBP >140 mmHg); insufficient quality of echocardiography. At baseline (n = 74) and at the follow-up visit (n = 46) cardiac imaging using cMRI and echocardiography techniques as described below was performed in all patients. In addition, standard clinical data were obtained from clinical examination in the context of our standard clinical investigation program for Fabry patients. Non-invasive measurement of blood pressure was performed manually three times on two different days using a standardized protocol. Prospective follow-up examinations (including cMRI and echocardiography) were performed at the next visit, resulting in a mean observation period of 51 ± 24 months.

According to the Declaration of Helsinki, written informed consent of all patients or their guardians was obtained. Ethics committee University of Würzburg approved this study. As it is a rare disease part of the patients were already included in other studies. [3, 7, 20]

### Laboratory measurements

N-terminal propeptide of brain natriuretic peptide (NT-proBNP) was determined as an easy applicable marker for cardiac dysfunction. In order to register early stages, NT-pro-BNP instead of brain natriuretic peptide (BNP) was used due to its higher sensitivity in patients with slight dysfunction. We abstained from using atrial natriuretic peptide (ANP) because of its restricted clinical relevance, mostly caused by the low biochemical half-life of only 3 minutes. NT-proBNP was measured with electro chemoluminescence immunoassayassay in our routine laboratory (coefficient of variation <7%).

### Magnetic resonance imaging and assessment of fibrosis

CMRI was performed after intravenous injection of gadopentetate dimeglumine 0.2 mmol/kg (Magnevist®, Bayer Schering Pharma AG, Berlin, Germany)on a 1.5 Tesla scanner (Magnetom Symphony Quantum, Siemens AG Healthcare Sector, Erlangen, Germany). Pictures were acquired with segmented inversion-recovery turboFLASH sequences (8 mm slice thickness, no slice spacing, breath hold technique, field of view 240 x 320 mm², matrix size 165 x 256, repetition time 7.5 ms, echo time 3.4 ms, flip angle 25° and acquisition window 250ms). The time of inversion (TI) was set to null normal myocardium (range 230-330ms). All consecutive short axis slices were used for manually tracing the area with pathological mid- or transmyocardial LE. The sum of areas was multiplied with the slice thickness and then set in relation to LV myocardium volume.

### Echocardiographic measurements

LV parasternal long axis images with M-Mode echocardiography (Vivid 7 [3.5 MHz], GE Vingmed Ultrasound AS, Horten, Norway) were used to measure end-diastolic and end-systolic dimensions (LVDD, LVSD) and thickness of the interventricular septum (IVST) and LV posterior wall (LVPWT). Additionally, left atrial (LA) diameter and the diameter of the aortic root were measured. By application of Simpson’s formula, LV ejection fraction (EF) was calculated based on 2- and 4-chamber view. Blood-pool pulsed Doppler of the mitral valve inflow was used to quantify the ratio of early-to-late (E/A) diastolic flow velocity and the deceleration time. The transmitral flow was determined by placing the Doppler window between the tips of the mitral valve leaflets and peak flow velocities in early (E wave) and late (A wave) diastole were measured. In case the E/A ratio was insufficient to quantify diastolic function, the difference between duration of blood flow in pulmonary veins and duration of blood flow in the left ventricle (both after atrial contraction) was calculated, indicating elevated filling pressure with values >30 ms. Additionally, tissue Doppler was performed for measuring the ratio between early transmitral flow and peak early tissue Doppler velocity (E/e’). Measurements of movement of septal and lateral mitral ring were averaged over three cycles. Using those parameters, diastolic function was graded either as normal, relaxation abnormalities (grade 1), pseudonormal (grade 2) or restrictive (grade 3). [16]

The global LV geometry can be quantified by the 3D sphericity index (SI). It is based on calculating how much the overall shape (which is assumed elliptic under normal circumstances) deviates from a sphere. Therefore, the volume of a virtual globe (4/3 * π * r^3^) is calculated with half the longitudinal diameter of the heart as the radius. This volume is then compared to the end-diastolic volume calculated by Simpson’s formula. The closer this index approximates the value “1.0” the better the LV shape resembles a spherical structur. SI values lower than 0.25 are considered normal. For patients after myocardial infarction a sphericity index of larger than 0.25 was suggested as a sign of myocardial alteration. [12, 16]

Standard apical views of the LV were acquired for off-line quantification of myocardial deformation (strain) by two-dimensional speckle tracking using ECHO-Pac Software (GE Vingmed Ultrasound AS, Horten, Norway). After manual selection of the region of interest (ROI), the myocardium was tracked, and tracking accuracy was confirmed by the reader. By semiautomatic postprocessing, longitudinal systolic strain of the 17 LV segments was extracted. Global strain was calculated as the mean value of all valid segments. Intra- and interobserver variability of longitudinal strain, was 9.8% and 11.4%, respectively, for single segments as previously described in our laboratory. [21] Using the same approach, intra- and interobserver variability for global strain was 6.5% and 7.7%, respectively. The analyses were performed in 25 patients with Fabry disease by two observers two times each, blinded for disease progression and patient’s data. Patients were selected randomly. The absolute magnitude of the differences was considered for analysis.

### Staging and disease progression

For staging of the severity of the cardiomyopathy, patients were grouped according to the amount of late enhancement (LE) in the LV myocardium. A value at baseline of ≥2% of LE positive myocardium (representing the 3^rd^ quartile of the amount of fibrosis among patients with pathological LE) was chosen to compare patients with mild and severe LE. At baseline, 39 patients showed no LE (ie, early stage cardiomyopathy), 23 mild LE (ie, intermediate stage) and 12 patients severe LE (ie, advanced stage).

As we know from previous publications [7, 20] the amount of fibrosis is of relevance for cardiac function and important for the clinical outcome (Malignant ventricular arrhythmias).

In addition, to compare the temporal progression of the cardiomyopathy between subjects, the annual increase of LE was calculated. Groups were then compared dichotomized at the median, ie, 0.2% LE increase per year thus separating slow from fast progression.

### Data analysis

Values are presented as mean ± SD, median with 25^th^ and 75^th^ quartile, or absolute numbers (percent), as appropriate. Correlation were expressed by Pearson´s or Spearman´s correlation coefficient, as appropriate. Differences between paired and unpaired groups were tested using t-test, Mann-Whitney U-test or Wilcoxon rank sum test, as appropriate. For categorical data, Chi-squared test or Fisher’s exact test were used. All differences were tested two-sided. Higher numbers of groups were tested using two-way ANOVA. Receiver operating characteristic analysis (ROC) as well as multivariable logistic regression analysis was performed to evaluate the ability of different values to distinguish between patients with different amounts of LE. P values <0.05 were considered statistically significant. Data was analyzed using SPSS version 21.0 (SPSS Inc, Chicago, IL).

## Results

The clinical characteristics of the study sample are presented in Table 1, both for the total cohort and in strata of the stage of cardiomyopathy. Patients with intermediate and advanced cardiomyopathy were older and presented with a higher systolic and diastolic blood pressure at baseline.

**Table 1:** Baseline characteristics of study participants in the total sample and stages of Fabry cardiomyopathy.

	Total	Early stage	Intermediate stage	Severe stage	P-value (ANOVA)
**Clinical characteristics**					
N (baseline)	74	39	23	12	-
Age (years)	36 ± 12	32 ± 13	37 ± 8	46 ± 10 † ‡	0.01
Female [n (%)]	45 (61)	27 (69)	13 (56)	5 (42)	-
N (follow-up)	46	17	19	10	-
Follow Up (months)	47 (29–67)	42 (24–59)	47 (30–67)	43 (30–73)	0.95
Height (cm)	171 ± 9	169 ± 8	171 ± 6	173 ± 11	0.96
Weight (kg)	68 ± 14	67 ± 13	69 ± 15	73 ± 16	0.32
Heart rate (bpm)	67 ± 12	68 ± 11	66 ± 12	67 ± 14	0.84
SBP (mmHg)	118 ± 15	115 ± 13	121 ± 16*	125 ± 19‡	0.04
DBP (mmHg)	80 ± 11	78 ± 11	81 ± 10	84 ± 12‡	0.16
a-Galactosidase activity (nmol/mg*mL)	0.14 (0.02–0.26)	0.17 (0.02–0.29)	0.13 (0.17–0.21)	0.12 (0.15–0.20)	0.05
**Medication** [n (%)]					
AT/ACE-inhibitors	8 (11)	1 (2.6)	4 (17)*	3 (25) ‡	<0.001
ß-blockers	5 (6.8)	2 (5.1)	1 (4)	2 (17)	0.41
Calcium antagonists	1 (1.4)	0	1 (2.9)	0	0.47
**Kidney**					
GFR (DTPA) (mL/min)	108 (64–132)	102 (86–133)	112 (100–137)	90 (56–119)	0.87
Proteinuria [n (%)]	13 (18)	4 (10)	6 (26)	3 (25)	-
Creatinine (mg/dL)	0.80 (0.61–0.92)	0.80 (0.60–0.91)	0.71 (0.62–0.85)	0.85 (0.74–1.21)	0.04
ACR (mg/g reatinine)	240±245	195±203	284±301	303±276	0.43
Kidney transplantation [n (%)]	0	0	0	0	-
Dialysis [n (%)]	0	0	0	0	-
**Cardiovascular**					
NYHA class [n (%)]					
I	54 (73)	33 (86)	16 (69)	5 (42) ‡	0.69
II	16 (22)	4 (11)	6 (26)	6 (50)	-
III	3 (4.1)	1 (2.6)	1 (4)	1 (8)	-
IV	0	0	0	0	-
NT-proBNP (mg/dL)	80 (39–844)	68 (39–79)	95 (27–290)	121 (55–844)‡	0.01
Hb (mg/dL)	13.6 ± 1.1	13.6 ± 1.3	13.5 ± 0.9	13.6 ± 0.9	0.97
**Pain** [n (%)]					
Neuropathic pain	43 (58)	20 (51)	13 (56)	10 (83)	-
Stress pain	30 (41)	14 (36)	11 (47)	5 (42)	-
Chronic pain	20 (27)	5 (13)	7 (30)	8 (67) ‡	-
Pain crisis	15 (20)	6 (15)	4 (17)	5 (42)	-
Frequent use of analgetics	14 (19)	4 (10)	6 (26)	4 (33)	-
**Neurological** [n (%)]					
Vertigo	21 (28)	9 (23)	5 (22)	7 (58) ‡	-
Tinnitus	28 (38)	10 (26)	12 (52)	6 (50)	-
Hearing loss	15 (21)	6 (15)	6 (26)	3 (25)	-
Depression	7 (9.5)	2 (5.1)	3 (13)	2 (17)	-
Cerebral insult	4 (5.5)	2 (5.1)	1 (4)	1 (8.3)	-
TIA	3 (4.1)	2 (5.1)	1 (4)	0	-
Dysarthria	0	0	0	0	-
**GI** [n (%)]					
Diarrhoea	29 (39)	11 (28)	9 (39)	9 (75) ‡	-
Gastric pain	22 (30)	8 (21)	10 (43)	4 (33)	-
Nausea	13 (18)	3 (7.7)	6 (26)	4 (33) ‡	-

Data in parenthesis are % of total

ACR, Albumine-creatinine-ratio

CM, cardiomyopathy

GI, gastrointestinal

TIA, transient ischemic attack

SBP, systolic blood pressure

DBP, diastolic blood presssure

AT, angiotensin

ACE, angiotensin converting enzyme

GFR, glomerular filtration rate

NYHA, New York Heart assosiation

NT-proBNP, N-terminal of brain natriuretic peptide

Hb, haemoglobin.

Significance at level 0.05 is indicated by

* for early vs. intermediate stage

† for intermediate vs. severe stage

‡ for early vs. severe stage

### Blood pressure measurement

In the total sample the mean SBP and DBP was 118 ± 15 mmHg and 80 ± 11 mmHg, respectively. However, SBP was higher in patients with myocardial fibrosis and depended on the stage of cardiomyopathy (early stage: 115 ± 13 mmHg; intermediate stage: 121 ± 16 mmHg; severe stage: 125 ± 19 mmHg; P = 0.047).

The mean SBP was highest in the group with a fast increase of replacement fibrosis, ie, >0.2% LE increase per year (n = 16; 125 ± 19 mmHg), compared to the group without such increase (n = 15; 117 ± 12 mmHg; p<0.05). Similarly, the subgroup with mild progression of LE showed higher SBP (n = 13; 122 ± 16 mmHg) than subjects without progression (p<0.05). There was also a trend for a gradient of higher diastolic blood pressures across these groups (early stage: 78 ± 11 mmHg; intermediate stage: 81 ± 10 mmHg; severe stage: 84 ± 12 mmHg; P = 0.161).

### Late enhancement imaging for the assessment of fibrosis

Among all patients, 35 (47%) showed mid- or transmyocardial LE (female 51%; male 58%; P = 0.12) with a mean volume of 1.69 ± 1.79% of LV mass. Male and female patients did not show significant differences in the amount of LE-positive volumes (18 women: 1.5 ± 1.4%; 17 men: 1.8 ± 2.2%; P = 0.25). Pathological LE was mainly restricted to the basal postero-lateral LV segments as described before. [22] Patients with LE-positive wall segments had experienced more cardiac events prior to study start compared to patients without LE (Table 2). Those findings have recently been published by our group. [7]

**Table 2:** Clinical history in the total cohort and according to stages of Fabry cardiomyopathy.

	Early stage		Intermediate stage		Severe stage	
	Baseline (n = 39)	Follow-up (n = 17)	Baseline (n = 23)	Follow-Up (n = 19)	Baseline (n = 12)	Follow-up (n = 10)
Bundle branch block	1 (2.6)	2 (12)	1 (4.3)	2 (10) *	2 (17)	4 (40) †
Cardiac arrhythmia	3 (7.7)	3 (18)	3 (13)	5 (26)	2 (17)	6 (60) †
Angina pectoris without CAD	0	1 (5.8)	0	2 (10) *	1 (8.3)	4 (40) †
ACVB, Stent or ICD	1 (2.6)	1 (5.8)	0	1 (5.3)	2 (17)	2 (20)

Mean observation period of 51 ± 24 months.

* p<0.005 intermediate CM baseline vs. intermediate CM follow-up

† p<0.005 advanced CM baseline vs. advanced CM follow-up

CAD, coronary artery disease

CM, cardiomyopathy

LE, late enhancement

ACVB, aorto-coronary vein bypass

ICD, implanted cardioverter defibrillator.

**Fig 1:**
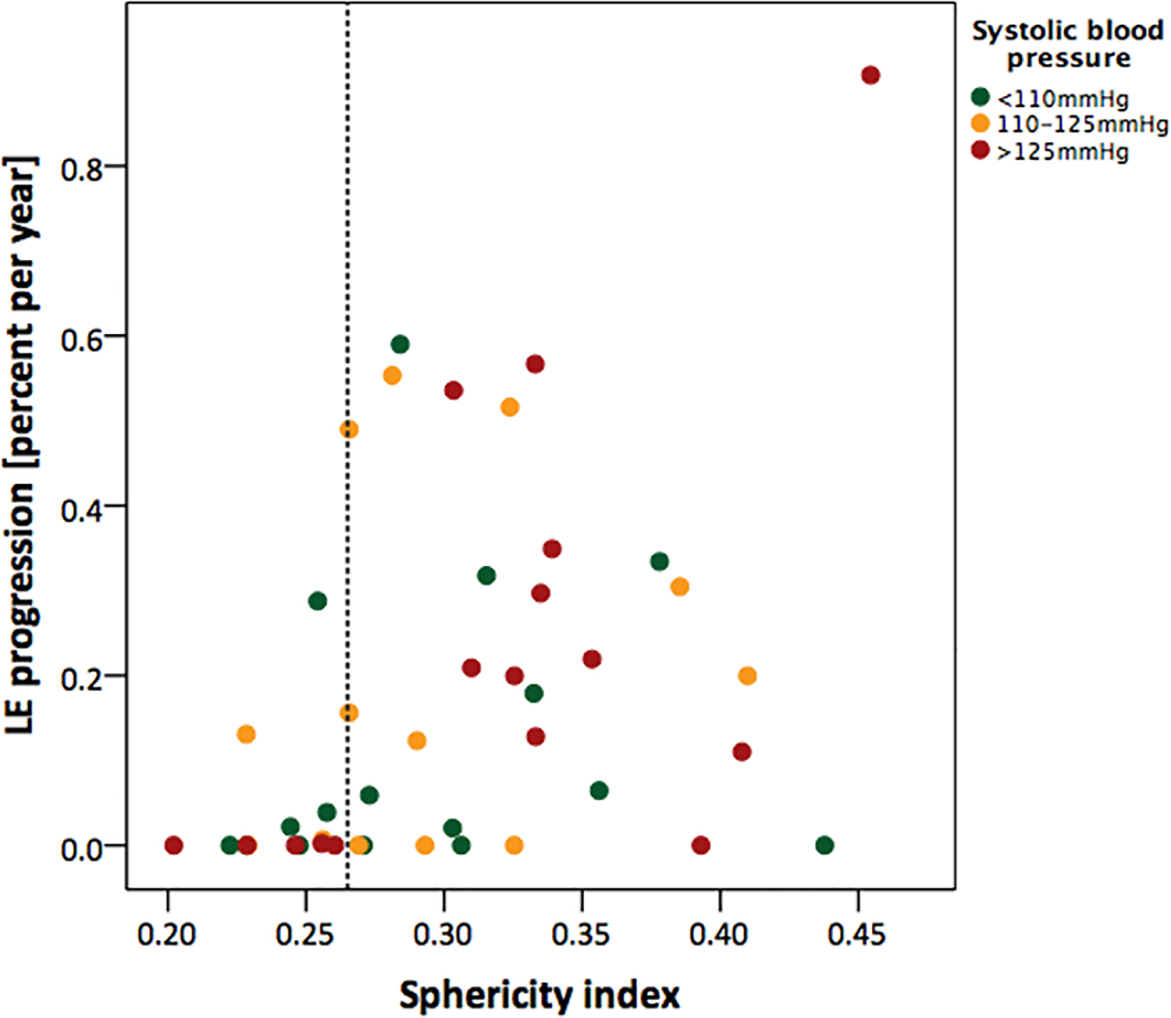
Scatter plot of annual progression rate of myocardial fibrosis versus the alterations in geometry. On the x-axis values of the sphericity index (SI, parameter for geometry) and on the y-axis the annual progression of late enhancement (LE, parameter for fibrosis) is displayed. The size of the red dots represents the systolic blood pressure (SBP) at baseline. The vertical line indicates the pathological value of the SI. Note the appearance of high SI in combination with elevated SBP and high progression rate. EDV; End-diastolic volume.

Those patients exhibiting LE already at baseline showed an increase of the total amount of LE-positive myocardium of 1.18 ± 0.30% during the follow-up period (baseline 1.7 ± 1.8%, follow-up 2.7 ± 2.2%; p<0.001). This amounted to a median increase of 0.23 (0.09–0.50). No patient showed a reduction of the amount of LE during follow-up. Two patients were LE-negative at baseline but developed new LE during follow-up. The progression of LE is shown in Fig 1. Only patients with LE had a significant increase of clinical events during the follow-up period (Table 2).

### Standard echocardiographic data

The results from the standard echocardiography divided in the different cardiomyopathy groups are presented in Table 3. The end-diastolic volume (EDV) increased with the cardiomyopathy stage (early stage: 76 ± 21 ml; intermediate stage: 85 ± 22 ml; severe stage: 97 ± 31 ml; P = 0.027). LA volume also numerically but not significantly increased with advanced stages (early stage: 32 ± 4 ml; intermediate stage: 33 ± 6 ml; severe stage: 36 ± 4 ml; P = 0.52). The baseline LV wall diameters also increased with advancing stages of the cardiomyopathy (IVST: early stage: 9 ± 2 mm; intermediate stage: 11 ± 2 ml; severe stage: 13 ± 2 ml; P<0.001). The baseline IVST was the only conventional parameter in echocardiography which showed a positive correlation with fast progression rate of myocardial LE (r = 0.381; P = 0.009). Valvular heart disease with more than mild affection was present in 5.3% of our patients and did not significantly differ between the groups.

**Table 3:** Echocardiography data at baseline and follow-up according to stages of Fabry cardiomyopathy.

	Early stage		Intermediate stage		Severe stage	
Variable	Baseline (n = 39)	Follow-up (n = 17)	Baseline (n = 23)	Follow-Up (n = 19)	Baseline (n = 12)	Follow-Up (n = 10)
Long. Diameter (mm)	82 ± 7	81 ± 9	79 ± 8	80 ± 8	81 ± 7	81 ± 11
Transv. Diameter (mm)	38 ± 6	36 ± 8	37 ± 5	37 ± 4	38 ± 5	40 ± 7
EDV (ml)	76 ± 21	75 ± 22	85 ± 22	86 ± 25	97 ± 31	91 ± 26
Width-length ratio	0.47 ± 0.07	0.44 ± 0.08	0.46 ± 0.06	0.47 ± 0.05	0.47 ± 0.06	0.48 ± 0.09
3D sphericity index	0.27 ± 0.06	0.28 ± 0.06	0.31 ± 0.06	0.33 ± 0.06	0.34 ± 0.05	0.34 ± 0.07
LVDD (mm)	48 ± 5	48 ± 3	48 ± 5	46 ± 5	49 ± 5	49 ± 4
LVSD (mm)	31 ± 4	32 ± 4	30 ± 5	30 ± 6	29 ± 5	33 ± 5 *
IVST (mm)	9 ± 2	9 ± 2	11 ± 2	10 ± 2	13 ± 2	12 ± 2
LVPWT (mm)	9 ± 2	9 ± 2	11 ± 2	10 ± 2	12 ± 2	12 ± 2
LA diameter	32 ± 4	33 ± 5	33 ± 6	33 ± 4	36 ± 4	36 ± 4
Fractional shortening (%)	37 ± 8	36 ± 5	37 ± 9	38 ± 9	40 ± 7	34 ± 10
EF (%)	62 ± 6	61 ± 7	62 ± 8	60 ± 6	64 ± 7	63 ± 9
E/A	1.4 ± 0.5	1.5 ± 0.6	1.4 ± 0.5	1.5 ± 0.9	1.2 ± 0.4	1.1 ± 0.3
DT (ms)	199 (181–218)	179 (139–214)	220 (183–260)	194 (162–226)	221 (186–248)	228 (162–284)
**Diastolic function**						
Normal	26 (67)	11 (64)	18 (78)	12 (63)	9 (75)	4 (40) *
Abnormal relaxation	5 (13)	2 (12)	4 (17)	2 (10)	1 (8.3)	1 (10)
Pseudonormal	8 (21)	2 (12)	1 (4)	5 (26)	2 (17)	5 (50)
Restriction	0	0	0	0	0	0

* p<0.005 intermediate CM baseline vs. intermediate CM follow-up

Long., longitudinal

transv., transversal

LVDD, left ventricular enddiastolic diameter

LVSD, left ventricular endsystolic diameter

E/A, early-to-late diastolic inflow ratio

DT, deceleration time

LA, left atrial diameter

IVST, interventricular septal thickness

LVPWT, left ventricular posterior wall thickness

EF, ejection fraction

EDV, end-diastolic volume

FS, fractional shortening.

LV mass was increased in advanced stages of the disease (early stage: 64.3 ± 18.3 g/m^2^; intermediate stage: 80.7 ± 25.9 g/m^2^; severe stage: 84.3 ± 21.4 g/m^2^; p<0.01).

Diastolic function and EF did not differ between the cardiomyopathy groups at baseline. Comparison of baseline and follow-up data is presented in Table 3.

### Morphological assessment by 3D sphericity index

The mean sphericity index in the Fabry cohort was 0.29 ± 0.06. Women had lower SI values (n = 45; 0.28 ± 0.05) compared to men (n = 29; SI 0.32 ± 0.07; p = 0.005). Patients in advanced stages of Fabry cardiomyopathy showed higher SI values compared to patients without cardiomyopathy (early stage: 0.27 ± 0.06; intermediate stage: 0.31 ± 0.06; severe stage: 0.34 ± 0.05; P<0.001). The same significant difference was detected when analyzing both genders separately (males: early stage: 0.29 ± 0.07; intermediate stage: 0.32 ± 0.08; severe stage: 0.35 ± 0.06; P = 0.026; females: early stage: 0.25 ± 0.04; intermediate stage: 0.30 ± 0.05; severe stage: 0.33 ± 0.04; P = 0.001).

We performed analysis of SI in 20 healthy controls (50% female, age 35±18 yrs). This resulted in mean SI of 0.24±0.01 with no difference between gender (p = 0.56).

**Fig 2:**
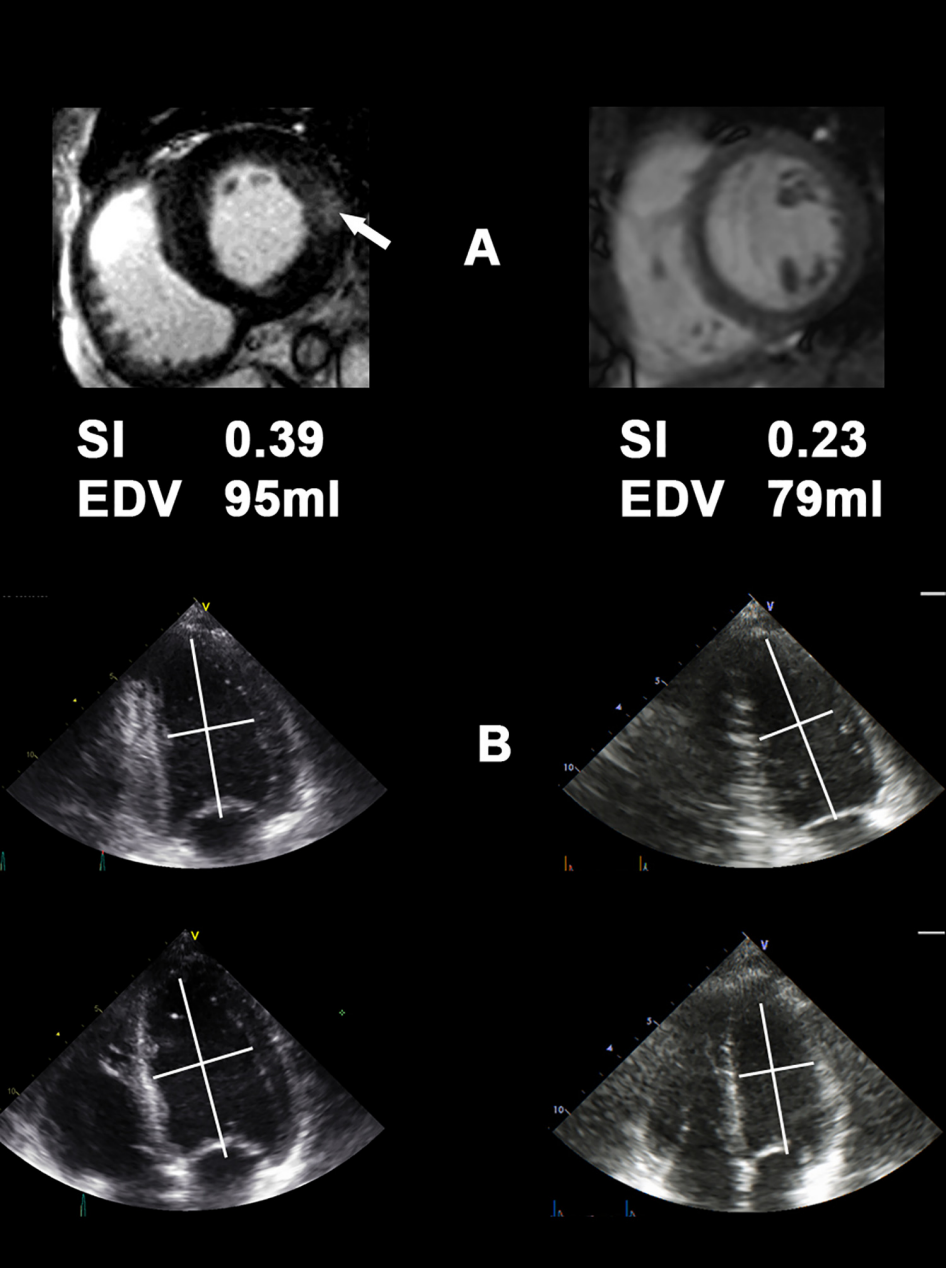
Typical cardiac magnetic resonance imaging (cMRI) (A) and echocardiography (B) of patients with normal sphericity index (SI; right row) and elevated SI (left row). Note the pathological late enhancement (LE; white arrow) in the cMRI and the more spherical shape of the LV in the left row. SI; sphericity index, EDV; end-diastolic volume.

A positive correlation was found between the amount of LE-positive myocardium and baseline SI (r = 0.49; p = 0.003) suggesting a (possibly direct) link between worsening sphericity and advancing stages of Fabry cardiomyopathy. Examples of typical echocardiographic images are shown in Fig 2.

In general, patients with a faster progression of fibrosis during follow-up presented higher SI values at baseline. The subgroup without increase of LE (n = 15) had baseline values of 0.27 ± 0.06. The subgroup with mild increase of LE (<0.2% per year; n = 15) presented with a SI of 0.29 ± 0.05 at baseline (p>0.001). The highest SI was found in the subgroup with fast increase of LE (≥0.2% per year; n = 16) with 0.32 ± 0.05 (P = 0.04).

In addition, patients with elevated SI (SI ≥ 0.25) at baseline showed higher SBP compared to the subgroup with normal SI (normal SI = 114 ± 13 mmHg; high SI = 122 ± 17 mmHg; p = 0.02).

At baseline, no patient received ERT. The follow-up was performed after 51±24 months. No significant difference for the change in SI was found between subjects with (n = 20) and without ERT.

To quantify the dependency of various independent variables with fast progression of fibrosis, univariate regression analysis was performed for SI (p = 0.011), SBP (p = 0.047), IVST (p = 0.021), LA (p = 0.025), LVEDD (p = 0.89), EDV (p = 0.28), EF (p = 0.31) and FS (p = 0.71).

**Fig 3:**
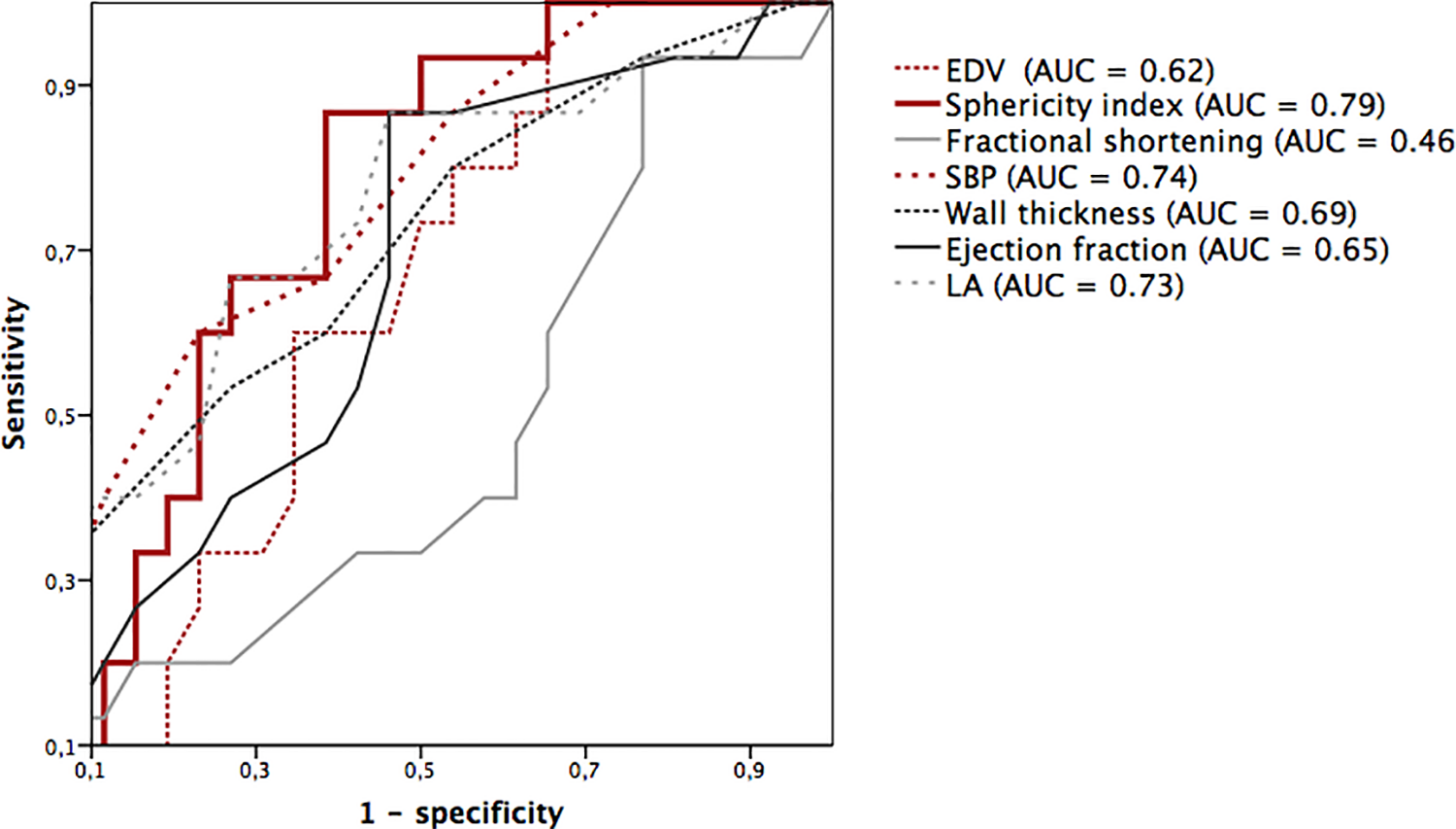
ROC curves of different echocardiographic parameters to identify patients with fast progression of late enhancement (LE). Parameters in the legend are ordered by the AUC. EDV, End-diastolic volume; EF, Ejection fraction; FS, Fractional shortening; LA, Left atrial diameter; SBP, Systolic blood pressure; SI, Sphericity index.

Variables with significant results were included in a multivariate logistic regression analysis adjusted for age and sex showed SI as the parameter to predict rapid progression of LE (Table 4). Receiver operating characteristic (ROC) analysis for the same parameters was performed. This analysis suggests that the SI is the most powerful predictor for fast increase of myocardial fibrosis (AUC = 0.785; P<0.05) (Fig 3 and Table 4).

**Table 4:** Multivariate logistic regression analysis for correlation with rapid progression of late enhancement.

Variable	OR (95% CI)	P-value	Wald	AUC
Elevated SI	10.38 (2.91–37.01)	<0.001	13.02	0.79 (0.69–0.90)*
SBP	1.45 (1.06–1.99)	0.02	6.68	0.74 (0.59–0.90)*
LA	1.22 (1.02–1.46)	0.03	4.78	0.73 (0.56–0.89)*
IVST	1.73 (1.07–2.80)	0.25	5.02	0.69 (0.52–0.82)*
NT-proBNP	1.00 (0.99–1.00)	0.05	3.75	0.64 (0-53-0.78)*
EF	1.2 (0.99–1.46)	0.07	3.32	0.65 (0.47–0.81)*
FS	0.90 (0.79–1.03)	0.12	2.39	0.46 (0.28–0.64)
EDV	0.98 (0.94–1.02)	0.28	1.15	0.62 (0.45–0.79)
Age	Fixed adjustment			0.59 (0.47–0.72)
Sex (female)	Fixed adjustment			0.42 (0.28–0.55)

SI, sphericity index

SBP, systolic blood pressure

LA, left atrial diameter

IVST, interventricular septal thickness

EF, ejection fraction

EDV, end-diastolic volume

FS, fractional shortening

NT-proBNP, n-terminal propeptide of brain natriuretic peptide

OR, odds ratio

CI, confidence interval

AUC, area under the curve

* p-value <0.05 for ROC-analysis.

During the follow-up period, two patients developed new myocardial replacement fibrosis. The mean SI at baseline from both patients was 0.32 ± 0.02. In addition, these two patients presented with higher SBP at baseline (121 ± 2 mmHg). In contrast, patients without fibrosis at baseline and during follow-up had lower SI values (0.27 ± 0.07) and lower SBP (113 ± 15 mmHg). While this additionally underscores the relation of SI with progression and development of fibrosis, no statistical test was performed due to the small sample size in this subgroup (n = 2).

The use of Angiotensin- (AT) or Angiotensin-Converting-Enzyme (ACE)-blockers was not significantly different in the subgroups.

### Myocardial deformation imaging

Global peak systolic strain as a parameter for global LV function was not significantly different between patients with normal and elevated SI (global peak systolic strain: normal SI: -18 ± 3%; elevated SI: -17 ± 3%; p = 0.351). In contrast, regional systolic strain in the basal postero-lateral, where replacement fibrosis is usually located, was significantly decreased in patients with elevated SI (-15 ± 4% in patients with an SI lower than 0.25 (n = 32) and -11 ± 7% in patients with elevated SI; n = 42; p = 0.025). The lowest systolic strain in postero-lateral wall segments was found in patients with rapid increase of LE (-6 ± 10; p = 0.025).

### Serum NT-proBNP-levels

NT-proBNP levels were positively correlated with the severity of the cardiomyopathy (early stage: 68 (39–79) pg/ml; intermediate stage: 95 (27–290) pg/ml; severe stage: 121 (55–844) pg/ml; ANOVA P = 0.009 with logarithmic transformation). Higher NT-proBNP levels were found in patients with an elevated SI (86 (51–520) pg/ml) compared to patients with normal SI (49 (33–158) pg/ml; P = 0.02). In the subgroup with fast increase of replacement fibrosis, higher NT-proBNP levels were found than in patients with slow and without increase of fibrosis (fast increase: 170 (58–361) pg/ml; slow increase: 86 (31–180) pg/ml; no increase: 62 (39–90) pg/m; P<0.001, respectively; ANOVA P = 0.311).

## Discussion

The current study shows for the first time the impact of LV geometry and blood pressure on both the stage at presentation and the adverse progression of the cardiomyopathy over time in a larger, well-characterized Fabry cohort. The main findings were: 1) The presence of myocardial replacement fibrosis assessed by LE imaging seems to determine the future accelerated progression of the cardiomyopathy. 2) The LV geometry is altered in Fabry cardiomyopathy, attaining an increasingly spherical shape. The extent of sphericity is positively related to the stage of the cardiomyopathy, thus predicting the progression of the disease. 3) Although (baseline) blood pressure was not markedly increased in patients with FD, a slightly elevated SBP was associated with an unexpectedly adverse impact on the cardiomyopathy. SBP is therefore closely related to alterations of the geometry and morphology of the myocardium and may–directly or indirectly–contribute to disease progression. 4) Levels of NT-proBNP are increased in relation to the stage of the cardiac involvement and can thus be used as a biomarker for the severity of the cardiomyopathy.

### Morphological features in Fabry cardiomyopathy

The early affection of myocardial function, even in absence of myocardial replacement fibrosis has recently been shown [7, 23]. Our study additionally links the geometry of the Fabry heart with the underlying morphology of the LV myocardium and the stage of the cardiomyopathy. Geometry was judged employing routine LV diameters, the volume of the LV chambers, and the SI. We found that a typical “early Fabry heart” is non-hypertrophic with normal LA and LV volumes and has a normal, ie, small ellipsoid shape. In contrast, the advanced cardiomyopathy is characterized by hypertrophy, volume increase of both the LA and LV, as previously described [24], increased wall stress as expressed by a raise in NT-proBNP, and gradual adoption of a more spherical LV shape. Thus, the current data confirm previous studies that the NT-proBNP level is related to the stage of the CM. [25, 26] The latter alterations can be easily detected and quantified by the SI, but not by the commonly used global parameters for systolic and diastolic LV function. The only conventional echocardiographic parameter associated with a faster progression of LE was the IVST, which showed a positive but weak correlation. The reasons for the changing shape of the Fabry heart during disease progression are unclear but may be attributable to an interaction of intrinsic and extrinsic factors. Likely candidates for intrinsic myocardial factors are the storage of globotriaosylceramides, the induced cellular hypertrophy and myocardial fibrosis. The role of extrinsic factors is less well defined, but might include the subtle afterload changes because of the Fabry vasculopathy [27], thus mediating a slightly increased blood pressure that may influence the course of the cardiomyopathy. Myocardial cells with α-galactosidase A deficiency seem to be more vulnerable for extrinsic influences.

While the blood pressure was always assumed an irrelevant factor in FD [19], the current data showed that already minor increases in SBP were strongly associated with an adverse course of the disease. Whereas this finding emphasizes the importance of SBP as a prognostic factor, our study was not suited to dissect out the role of SBP as a causal pathogenetic factor. It is conceivable, however, that an elevated SBP induces a shape change as well as an increase in wall stress in the already damaged and thus vulnerable segments of the hearts, thus accelerating fibrosis progression. This vulnerability may render the Fabry heart particularly susceptible to minor increases in blood pressure, ie, increases that are usually well tolerated in the healthy heart. According to the law of Laplace a blood pressure induced increase in afterload is particular relevant in the basal part of the heart where the curvature of the LV is relatively flat. [15] Consistently, myocardial fibrosis is typically located in the basal LV segments. [11] An increase in SI may be viewed as a compensatory mechanism aiming towards lowering local wall stress. However, it is still unclear why especially the basal postero-lateral wall is affected by the replacement fibrosis, whereas other basal segments are rarely affected.

All factors discussed above progressively stress the Fabry heart, increase enddiastolic pressures and thus NT-proBNP levels, and will ultimately cause heart failure.

Another important factor, regarding the heart in FD, is the influence of impaired kidney function on blood pressure, LE and altered volume relations, which could have several affections on the heart. To illustrate all these effects a much larger number of patients is necessary, which very difficult in Fabry disease.

### Incident fibrosis in FD

The two patients who were free of replacement fibrosis at baseline but developed LE during follow-up are particularly interesting. As they were not hypertrophic and free of replacement fibrosis they were classified as “early cardiomyopathy” at baseline. However, both patients had an increased SBP and SI, which might have triggered the relatively fast progression towards a more advanced cardiomyopathy stage with replacement fibrosis. These findings are important for three reasons: First, it confirms our hypothesis that the geometry and the blood pressure are important factors for the progression of the cardiomyopathy. Second, they suggest that an altered LV geometry is not necessarily secondary to myocardial replacement fibrosis but actually might be an early indicator for subsequent development of fibrosis. Third, when a Fabry patient is followed clinically, even a slight increase of blood pressure may indicate the susceptibility for an accelerated progression. It needs to be shown, however, whether a targeted treatment of SBP in these subjects might slow or halt progression.

**Fig 4:**
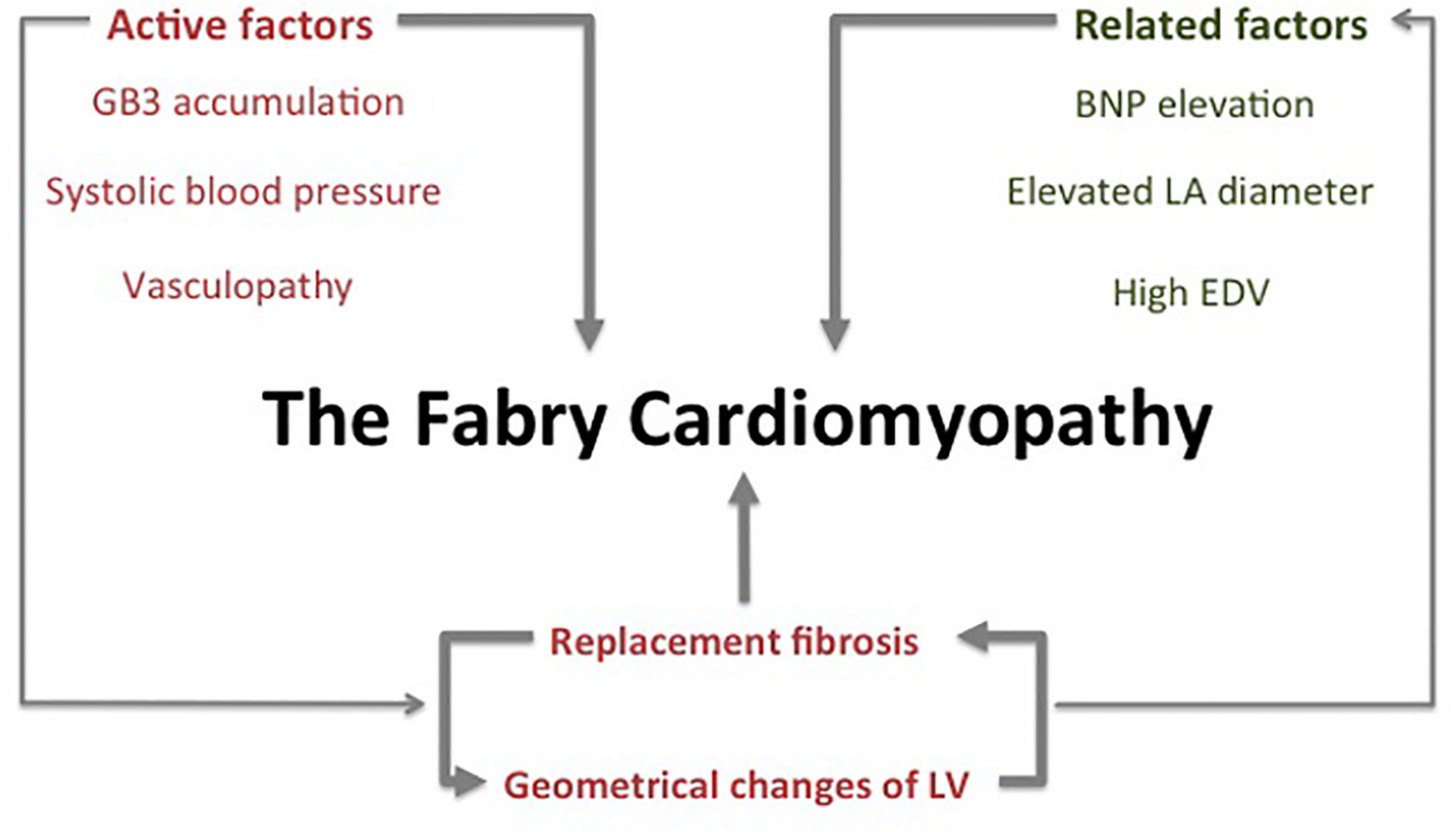
Overview of presumed factors and their interrelation influencing the progression of Fabry cardiomyopathy. BNP, Brain-natriuretic peptide; EDV, End-diastolic volume; GB3, globotriaosylceramide; LA, Left atrium; LV, Left ventricle.

Therefore, all these factors are influencing each other and affect the progression of the CM, as shown in Fig 4. [28, 29, 30, 31]

### Clinical impact

Echocardiography and cardiac MRI are part of the current standard clinical protocol for the assessment of Fabry cardiomyopathy. Not only the current stage of the disease, but also an estimate of the future progression of the cardiomyopathy are clinically important. From the presented data the recommendation might be derived not only measure the established parameters like LE (by cMRI) and end-diastolic wall thickness (IVST, LVPWT; by cMRI or echocardiography), but also to observe morphological features like the SI. The SI is simple to measure with most echo machines and values can be reliably compared across repetitive follow-up examinations.

Prediction of fast progression of fibrosis will not be possible with one single measurement or one single parameter. Thus, physicians working with Fabry patients should be aware of changes in those parameters during disease progression, indicating a higher fibrosis progression rate.

In all Fabry patients, independent of the stage of the disease, the blood pressure should be adequately controlled. Thus, a 24-hour blood pressure measurement should be done routinely and oral medication with, i.e., renin-angiotensin-aldosterone-system (RAAS) blockers should be considered. These substance classes have the advantage, to positively influence SBP (enhance unloading), cardiac remodeling (prevent/slow cardiac fibrosis) and the kidney function (decrease proteinuria). [18, 31, 32] However, the prognostic benefit of a targeted blood pressure treatment has to be investigated in dedicated controlled trials.

The current data is in line with the view that the development of the Fabry cardiomyopathy is an ongoing process as soon as changes in geometry and myocardial fibrosis are present. This suggests that very early treatment ERT before these markers can be detected might be preventive. The collection of data in a randomized controlled trial supporting this notion constitutes probably one of the most urging issues of the Fabry research agenda.

### Study limitations

Because of ethical reasons, myocardial biopsies could not be obtained for definite proof of fibrosis. However, this technique allows to sample only selected amounts of tissue, thus not allowing to quantify the amount of fibrosis.

The reported blood pressure data result from isolated measurements in hospital setting. The results would be more meaningful with ambulatory Holter blood pressure measurements, which could not be performed in this study.

SI was significantly different according to gender. A possible explanation is the higher age of male patients in our cohort and the fact that male patients are usually more affected in this x-chromosomal disease.

Another limitation is the fact that the impact of treatment with i.e ACE inhibitors and also ERT cannot be evaluated in detail. The influence of other factors of impaired kidney function on fibrosis and blood pressure could not be eliminated

## Conclusions

The current study is focusing on new parameters, relating to and influencing on the Fabry CM. The investigation of these parameters (late enhancement, sphericity index and blood pressure) is helpful for the understanding of the development of the cardiomyopathy. Myocardial cells with enzyme deficiency tend to be more vulnerable for extrinsic influences like slightly increased blood pressure. Left-ventricular geometry is altered in relation to the stage of the cardiomyopathy. These alterations can be easily detected by repetitive echocardiographic measurement of the sphericity index, which is predicting progression of the disease.
